# Supplementary material for: Molecular Mechanism of Small-Molecule Inhibitors in Blocking the PD-1/PD-L1 Pathway through PD-L1 Dimerization
Source: Int J Mol Sci. 2021 Apr 30;22(9):4766. doi: 10.3390/ijms22094766 (PMC8125578; doi:10.3390/ijms22094766)
Supplement: Supplementary file 1 [file ijms-22-04766-s001.zip › ijms-1177930-supplementary.pdf]

Supplementary Materials

Molecular Mechanism of Small-molecule Inhibitors in Blocking the PD-1/PD-L1 Pathway through PD-L1 Dimerization

Yan Guo, Yulong Jin*, Bingfeng Wang, Boping Liu*

Key Laboratory for Bio-based Materials and Energy of Ministry of Education, College of Materials and Energy, South China Agricultural University, Guangzhou 510630, People's Republic of China; guoyan@stu.scau.edu.cn (Y.G.); wbfeng@scau.edu.cn (B.W.)

* Correspondence: jyl@scau.edu.cn (Y.J.); boping@scau.edu.cn (B.L.)

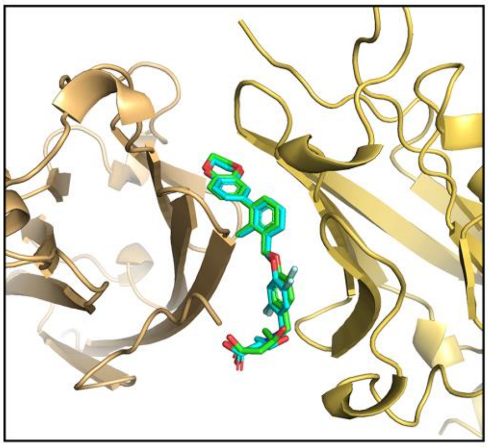

Figure S1. Overlap of the vina-docked pose and native conformation from the crystal structure (PDB ID: 5N2F). The docked pose and native confirmation are coloured cyan and green, respectively. The APD-L1 and BPD-L1 are coloured orange and yellow, respectively.

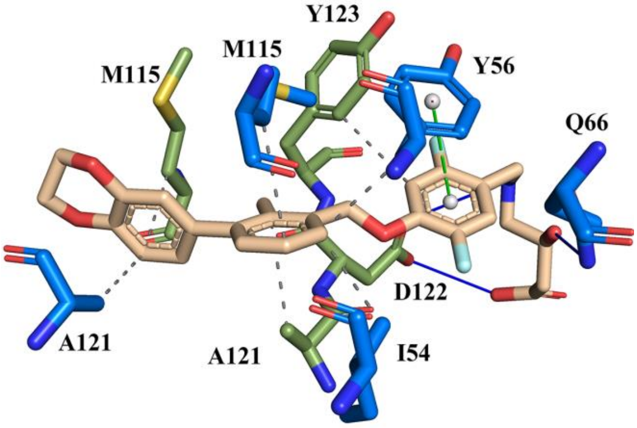

Figure S2. Binding modes and interactions between the docked BMS-200 and PD-L1 dimer. The APD-L1 and BPD-L1 are coloured green and blue, respectively. BMS-200 is coloured beige. The H bond, hydrophobic and Π-stacking interactions are also shown.
